# The precision principle: driving biological self-organization

**DOI:** 10.3389/fnetp.2025.1678473

**Published:** 2025-11-12

**Authors:** Raymond Roy, Kiranpreet Sidhu, Gabriel Byczynski, Amedeo D’Angiulli, Birgitta Dresp-Langley

**Affiliations:** 1 Department of Neuroscience, Neuroscience of Imagination, Cognition, and Emotion Research Lab, Carleton University, Ottawa, ONT, Canada; 2 Chlildren’s Hospital of Eastern Ontario Research Institute, Ottawa, ONT, Canada; 3 Department of Clinical Neurosciences, University of Geneva and Geneva University Hospitals, Geneva, Switzerland; 4 Centre National de la Recherche Scientifique, Strasbourg University, Paris, Strasbourg, France

**Keywords:** self-organization, brain, evolution, neural circuits, neural learning, precision, plasticity, networks

## Abstract

In this perspective, we introduce the *Precision Principle* as a unifying theoretical framework to explain self-organization across biological systems. Drawing from neurobiology, systems theory, and computational modeling, we propose that precision, understood as constraint-driven coherence, is the key force shaping the architecture, function, and evolution of nervous systems. We identify three interrelated domains: Structural Precision (efficient, modular wiring), Functional Precision (adaptive, context-sensitive circuit deployment), and Evolutionary Precision (selection-guided architectural refinement). Each domain is grounded in local operations such as spatial and temporal averaging, multiplicative co-activation, and threshold gating, which enable biological systems to achieve robust organization without centralized control. Within this framework, we introduce the *Precision Coefficient*, $P\left(z\right)=C\left(z\right)-\alpha R\left(z\right)$ , which formalizes the balance between network coherence and resource cost and serves as a simple quantitative outline of the principle. Conceptually, this formalism aligns with established learning mechanisms: Hebbian reinforcement provides the local substrate for weight changes, while winner-take-all and k-winners competition selectively eliminates weaker synapses, together increasing $C\left(z\right)$ and reducing redundancy within $R\left(z\right)$ . Rather than framing the theory in opposition to existing models, we aim to establish the *Precision Principle* as an original, integrative lens for understanding how systems sustain efficiency, flexibility, and resilience. We hope the framework inspires new research into neural plasticity, development, and artificial systems, by centering internal coherence, not prediction or control, as the primary driver of self-organizing intelligence.

## 1 Introduction

When most people think of a nervous system, they imagine vast webs of interconnected neurons shooting signals back and forth. This assumption raises an intriguing question: Do single-celled organisms have nervous systems?

If we define a nervous system by its ability to connect an organism to its external environment, allowing it to respond, adapt, and survive, then unicellular organisms arguably qualify. Certain bacteria and unicellular eukaryotes navigate their environment using molecular structures like flagella and microvilli (Lynch, 2024). Through chemical and physical sensors such as gated ion channels, these structures interact with the environment and initiate changes in the organism’s movement (Saimi et al., 1988). Rather than being directed by a central command, such reactions emerge from dynamic interactions between internal structures and external cues. Even single-celled life exhibits primitive forms of organization, sense integration, and environmental adaptation; these underlying mechanisms, rooted in life preservation and stimulus response, represent the early blueprint of nervous systems in complex organisms.

From this foundation, the principle of minimal, self-evoked structure scales upwards (Dresp-Langley, 2024). As organisms evolved nervous systems, their purpose remained constant, even as complexity increased; here, chaos gives rise to order. They integrate, prioritize, and represent the world (Wan et al., 2024) while adhering to the same logic: organisms self-organize not because they are told to, but because their survival demands it. In this perspective, we call this internal drive the *Precision Principle*, a field-unifying theory of constraint-driven coherence that governs how nervous systems organize, adapt, and endure across scales. We express this logic with a simple quantitative form, the *Precision Coefficient*, $P\left(z\right)=C\left(z\right)-\alpha R\left(z\right)$ , where $P\left(z\right)$ balances network coherence against resource cost and α≥0 tunes their relative weight. The coefficient provides a concise quantitative outline that links these domains without reducing them to a rigid optimization scheme, and the conceptual definitions that guide each domain follow.

**FIGURE 1:**
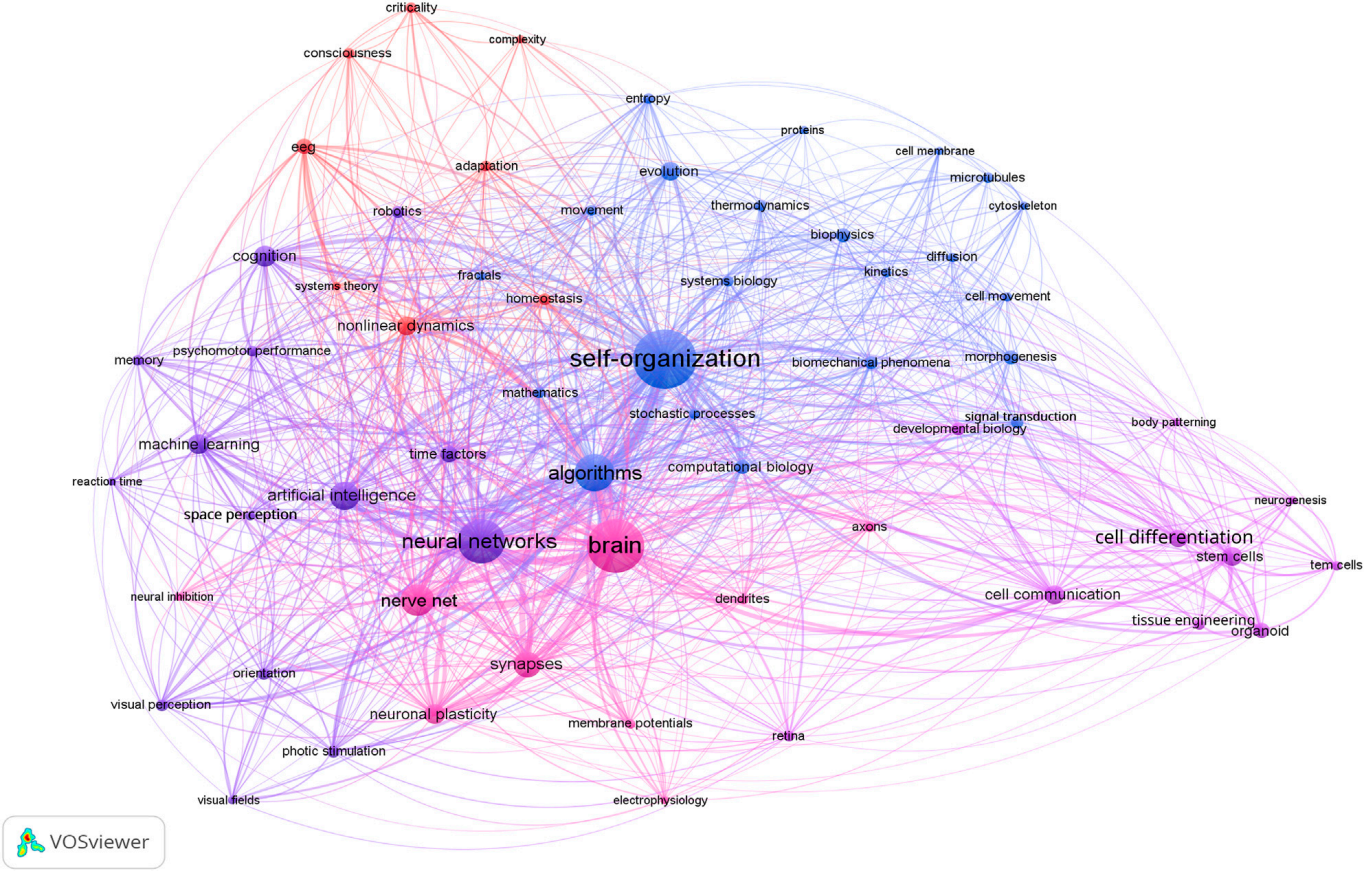
Conceptual landscape of self-organization across biological and artificial systems.

To underscore the scope and centrality of self-organization across disciplines, we propose Figure 1 above, which depicts a global co-occurrence network of key concepts extracted from 1,810 peer-reviewed articles on biological and artificial systems. Node size scales with term frequency and edge thickness with co-occurrence strength. At the heart of the network, “self-organization” exhibits the highest centrality and forms dense connections with a vast array of topics spanning thermodynamics, developmental biology, neural information processing, machine learning, robotics, and cognitive dynamics. Such prominence highlights how self-organization functions as a unifying principle, observed across physical, biological, and computational disciplines, to coordinate pattern formation, information flow, and adaptive behavior in systems characterized by *Precision* in the face of variability, the proper interpretation of which will be presented in the following section.

### 1.1 Entropy

The concept of entropy describes how isolated systems tend to evolve toward more probable states, which have a greater number of possible micro-configurations. This causes an increase in disorder or uncertainty. While the natural trend is for disorder to grow, living organisms keep their internal organization by exporting entropy to the outside environment, meaning they convert ordered energy into less useful, degraded energy.

Within a world that accelerates towards this effect under the 2^nd^ law of thermodynamics, we consider self-organizing forces in living systems, under the drive of evolution towards functional stability, as designed to counteract entropy. Self-organization allows for the creation of our nervous system and brain, coordinating billions of cells to interact with reality. In this paper, we propose that the brain’s capacity for self-organization and multimodular plasticity is guided by a fundamental internal drive towards what we term *Precision.*

### 1.2 Precision

Precision in this context may be seen as an emergent, constraint-driven optimization criterion that governs both the architecture of brain circuits and their functional dynamics, sculpting their structure and activity in ways that maximize efficiency while preserving internal coherence. In what follows, “precision” refers to emergent coherence under constraint rather than to fine-tuning or generic optimization, and we use the *Precision Principle* as a unifying framework to organize the levels of analysis developed here. Just as intermolecular forces (i.e., London Dispersion Effect) determine how molecules settle into stable configurations under physical constraints, biological systems similarly exploit such principles to drive toward precision in structure, functionality, and evolution.

Drawing on and expanding ideas from Hebbian Law, memory consolidation, cross-modal plasticity, and predictive coding, the *Precision Principle* offers an adjusted perspective from theories like the Free Energy Principle (FEP). While both the FEP and *Precision Principle* yield similar outcomes, namely, pattern recognition and internal coherence, the underlying mechanisms differ fundamentally.

The FEP characterizes cognition as a predictive engine constantly questioning “what’s next?”, inherently driven by minimizing surprise through alignment with external reality (Friston, 2010). A closely related account, Bayesian brain theory, likewise portrays cognition as probabilistic inference, combining prior beliefs with sensory likelihoods in a precision-weighted manner to produce posterior estimates (Knill and Pouget, 2004; Yuille and Kersten, 2006). However, such personification, whether of a brain that minimizes free energy or one that optimally updates posteriors, misrepresents biological reality; the biological brain and conscious mind are distinct entities acting in tight conjunction (D’Angiulli and Sidhu, 2025).

The *Precision Principle* challenges the centrality of surprise minimization (and ideal Bayesian optimality), proposing instead that cognitive coherence and pattern recognition emerge primarily from internal, self-organizing neurobiological processes. In this framing, cognition arises through intrinsic molecular interactions and neural dynamics rather than an external alignment. Indeed, surprise minimization itself can be considered an emergent property within our consciousness resulting from deeper biological tendencies toward efficiency and equilibrium within an ever-changing environment. But precision, not merely prediction, organizes the brain’s structural and molecular logic. Adding surprise minimization to intrinsic neural dynamics is analogous to projecting human morality onto the animal kingdom, such as rooting for a gazelle escaping from a lion; these moral narratives have no intrinsic validity outside human consciousness. Similarly, cognitive coherence is fundamentally a product of internal neural organization, which, while grounded in environment, is not constrained by it.

## 2 Precision-driven self-organization: a three-part classification

While differentiation delineates how neurons acquire distinct molecular and functional identities and specialization describes their progressive narrowing to a single computational role, Precision imposes an orthogonal constraint: circuits must not only exist but justify their persistence in the network’s economy. Thus, building on the foundational notion of Precision as constraint-driven structure, we propose that it manifests in three interwoven domains: *Structural Precision*, *Functional Precision*, and *Evolutionary Precision*.

Beyond its impact on individual circuits, Precision operates as a unifying principle across multiple timescales. Unlike theological or metaphysical invocations of design, this usage refers to biologically grounded rules (e.g., energy efficiency, redundancy minimization, metabolic optimization) that shape neural architecture through interaction, pruning and constraint, rather than the current *status quo* relating to foresight and or intention. Structurally, it generates compact, high-efficiency modules with minimal redundancy; functionally, it governs on-the-fly redeployment and robust buffering of circuits via gated short-term memory traces; and evolutionarily, it determines which functionally validated configurations become encoded in the genome under selective pressure. By embedding the same core rules across developmental, operational, and phylogenetic contexts, Precision offers a single, mechanistic framework explaining how the brain perpetually balances stability against adaptability in service of resilient, energy-efficient computation. This framework is further clarified in Table 1, which contrasts traditional definitions with their reinterpretation under Precision.

**TABLE 1:** Common definitions in the study of cell organization, and their key differences from precision.

Term	Traditional Definition	How precision differs
Differentiation	Process by which cells or neurons become specialized	Precision requires that differentiated circuits justify continued inclusion in the network
Specialization	Narrowing of functional role	Precision accounts for whether that function is retained, deployed, or pruned based on internal system efficiency
Prediction	Anticipation of external input	Precision reframes prediction as an outcome of internally optimized coherence, not a primary directive

### 2.1 Structural Precision: from local synapses to global network design

As previously stated, Structural Precision shapes modular architectures that minimize redundancy by promoting tight local clustering among co-activated neurons. It also establishes long-range pathways for distributed integration, an emergent pattern of modular connectivity grounded in unsupervised, activity-dependent learning (Dresp-Langley, 2020). Through long-range links, modules exchange information so that specialized circuits can operate independently while still integrating seamlessly into global computations. In such self-organizing networks, these circuit interactions are mirrored at the molecular scale, where structural modifications accumulate as long-term memory (LTM) traces along synaptic pathways (Milano et al., 2023). This coupling of local change and global coordination arises through unsupervised Hebbian learning: each synapse adjusts its weight solely from the timing of pre- and post-synaptic activity, without an external supervisor (Dresp-Langley, 2020). In this way, synaptic microstructure and global topology evolve together in a tightly coupled process. As Grossberg (1993) showed in his additive formulation below, (Equation 1), each LTM trace can be described by a differential equation with a selective gated-decay term that restricts plasticity to periods of significant postsynaptic activation:

$$\frac{{dz}_{ji}}{dt}=h_{j}\left(z_{j}\right)\left(-K_{ji}z_{ji}+L_{ji}f_{i}\left(z_{i}\right)\right)$$ (1)

where the gating function *h*_*j*_ *(z*_*j*_ *),* a thresholder sigmoid, ensures that only sufficiently strong postsynaptic responses permit plasticity, thereby capturing the balance between Hebbian growth and synaptic decay. Here, *z*_*ji*_ is the persistent component of synaptic strength from neuron *j* to neuron *i*, reflecting the accumulated history of neural interactions. The decay constant *K*_*ji*_ eliminates spurious potentiation, while the Hebbian term *L*_*ji*_ *f*_*i*_(*zi*) reinforces synapses in proportion to presynaptic activation *f*_*i*_ (*z*_*i*_) (Grossberg, 1993). Because the same decay–gating–potentiation rule applies across scales, tiny synaptic tweaks propagate outward to create from-local-to-global organization, where distant nodes link into coherent functional motifs (Dresp-Langley, 2020). At its core, circular causality, where local changes shape global structure, which in turn biases future local activity, underpins the brain’s ability to form scale-free hubs without centralized control.

Over developmental and evolutionary timescales, these same multiplicative co-activation and threshold-gating rules also govern how the network’s wiring diagram evolves (Knoblauch, 2017). Synaptic pathways that repeatedly co-activate across environmental inputs accumulate LTM weight, forming densely interconnected modules that mirror the statistical “shape” of those inputs, while weakly used connections decay and are pruned. For example, we can imagine a network repeatedly exposed to vertical edges in natural scenes: each time two neurons co-fire on a vertical contour, their connecting weight *z*_*ji*_ gets a tiny boost, but only if the postsynaptic cell itself was “engaged” thanks to the gating function *h*_*j*_ *(z*_*j*_ *).* Across thousands of such episodes, clusters of vertical-edge detectors self-assemble into densely wired modules analogous to cortical columns, while all other, less-used cross-links atrophy away, yielding an architecture so well-tuned to vertical features that it functions like a biologically evolved edge detector, analogous to orientation columns in visual cortex. A continual cycle of selective potentiation and pruning exemplifies dynamic system growth, allowing a fixed-size network to explore an exponential repertoire of functional states without adding neurons (Dresp-Langley, 2020). By valorizing only connections that improve system fitness and suppressing the rest, the brain dynamically reallocates resources to emerging computational niches. This balance between strengthening coherence and reducing redundancy directly anticipates the variables of the *Precision Coefficient*, $P\left(z\right)$ , which we develop more fully later in this section.

By enforcing the identical LTM update rule on every outgoing synapse, a local symmetry axis in which all synapses update identically based on local activity, the network not only converges stably, but it also gains the ability to incorporate new patterns without biasing older ones (Pena and Cerqueira, 2017). In practical terms, when novel inputs arrive (say, learning a new texture), Structural Precision machinery carves out fresh modules in parallel to existing ones, without corrupting or destabilizing existing feature maps. This symmetry-enforced balance between plasticity and stability stands as a hallmark of cortical development and underlies phenomena like map reorganization after sensory deprivation or injury, where the same constraint-driven coherence quietly shapes both adaptation and learning (Rabinowitch and Bai, 2016).

Therefore, Structural Precision is encoded in the very equations of LTM, where selective reinforcement and decay rules bias networks toward coherence under constraint. Cultured neural networks illustrate the same principle at a different scale: when left to self-organize, they form compact architectures that minimize metabolic cost while preserving representational power. Dense hubs emerge around high-utility features, while less informative connections recede into sparse links. These dynamics echo the formation of cortical columns and the evolvable architectures of sensory and motor maps across species. Thus, Structural Precision is not an abstract labeling but an intrinsic outcome of the very rules that govern synaptic change and network design.

### 2.2 Functional Precision: adaptive reconfiguration without structural overhaul

Functional Precision describes how existing processing modules are transiently redeployed in response to novel or missing inputs, preserving latent computational mappings even as their active functions shift. A critical mechanism underlying this flexibility is the brain’s use of short-term memory (STM) traces, rapidly evolving activation patterns that buffer contextual information without altering long-term synaptic weights (Johnson et al., 2013). Within this framework, each node’s STM trace $dz_{i}/dt$ evolves according to the generalized additive model originally proposed by Grossberg (1993) (Equation 2):

$$\frac{{dz}_{i}}{dt}=-az_{i}+\sum_{m}b_{im}S_{m}z_{m}+I_{i}\left(t\right)$$ (2)

Here, Functional Precision arises not from fixed architecture but through the dynamic regulation of transient neural activity. In this context, the decay term −*az*_*i*_ ensures that modules deactivate without sustained input, minimizing interference. At the same time, the co-activation term $\sum_{m}b_{im}S_{m}z_{m}$ enforces context-sensitive recruitment: only when modulatory signals *S*_*m*_ align with upstream activity *z*_*m*_ do downstream traces respond. Critically, this multiplicative interaction imposes a narrow operational window, ensuring that modules activate only under tightly tuned conditions, favoring relevant over spurious input. With the final term *I*_*i*_ *(t),* representing direct external drive, shifting demands can transiently repurpose circuits. A gated-trace mechanism like this underlies the brain’s adaptive ability, flexibly redeploying modules whenever inputs or modulatory cues shift (Dresp-Langley, 2020). Through tuning the decay constant and co-activation weights, the network can transiently amplify relevant circuits without altering its core synaptic map.

Rather than requiring structural rewiring, this framework supports conditional reuse: latent mappings are conserved, expressed only when internal and external signals converge, and decay naturally restores baseline functions once the drive subsides (Gillary et al., 2017). Accordingly, the equation does not model function directly, but rather the constraints that regulate functional access, capturing a reversible, selectively gated precision that avoids both rigidity and chaos. In this light, what emerges is the need for a compact expression that captures how coherence rises under constraint, with access tuned rather than prescribed. We later formalized this logic in the *Precision Coefficient*.

Empirical work illustrates how such constraint-driven coherence unfolds in real networks. In dissociated rat cortical cultures devoid of structured external input, neurons self-assemble into small-world functional networks within 4 weeks *in vitro*. One longitudinal study using planar microelectrode arrays reported a clustering coefficient that rose from approximately 1.2 at Day *In Vitro* (DIV) 14 to 2.3 by DIV35, while the small-world index surpassed 1.2 (*p* < 0.01), reflecting a transition from segregated modules to integrated hub-and-spoke architectures (Downes et al., 2012). According to transfer-entropy reconstructions, by DIV21 effective connectivity exhibits pronounced clustering and short path lengths characteristic of small-world topology (Orlandi et al., 2014). At the same time, spontaneous firing organizes into neuronal avalanches whose event-size distributions follow a power law with exponent α ≈ −1.5 across four orders of magnitude, indicating critical dynamics that maximize dynamic range and information throughput (Beggs and Plenz, 2003). In addition, rich-club analyses reveal the early emergence of highly connected hub neurons by DIV14, which consolidate by DIV28 to coordinate global network bursts (Hahn et al., 2010).

Crucially, functional reassignment depends on multiplicative co-activation and biologically gated thresholds. Potentiation of downstream targets via the term *b*_*im*_ *S*_*m*_ *z*_*m*_ occurs only when both pre- and postsynaptic STM traces exceed a biologically meaningful threshold θ ; subthreshold activations are effectively shunted, preventing trivial fluctuations from triggering reassignment (Sajikumar and Korte, 2011). In practice, this means that only suprathreshold co-activations drive functional redeployment, safeguarding the network against runaway or noisy rewiring.

Empirical studies in early-blind humans further provide compelling support for this STM-trace gating model. Sustained auditory or tactile stimulation can transiently raise STM traces in occipital areas, normally devoted to vision, above threshold, allowing those modules to without any structural rewiring (Rabinowitch and Bai, 2016; Kılınç et al., 2025; van der Heijden et al., 2019). For example, pitch discrimination by blind listeners activates V1 and surrounding cortex when prolonged auditory stimulation maintains STM traces long enough for functional recruitment (Pang et al., 2024), only to revert to baseline visual mappings upon removal of the drive.

More broadly, simple stimuli reshape primary cortices (e.g., V1) only when attention or task demands raise their STM traces above threshold, whereas higher-order areas such as V5/MT, with broader receptive fields and lower gating thresholds, can adopt new functions even during passive stimulation (Lewis et al., 2010). A similar form of gating logic builds in functional resiliency, as latent pathways can instantly compensate when primary circuits falter, preserving core computations under perturbation (Dresp-Langley, 2020). Since STM traces decay only when truly unused, transient disruptions never permanently silence the network’s critical routes.

Thus, STM-mediated gating, multiplicative co-activation, and threshold-gating jointly furnish Functional Precision’s reversible substrate (Hansel & Yuste, 2024). When particular circuits are repeatedly co-opted under stable demands, this reversible reuse gives way to functional plasticity, whose pathways gain lasting readiness without structural rewiring (Dresp-Langley, 2020).

This is further reinforced by the gradual lowering of gating thresholds for frequently engaged modules ensures that familiar tasks become ever more efficient yet remain reversible if contexts shift again. Building on that adaptability, a reversible form of co-option not only explains cross-modal plasticity in blindness but also exemplifies a general mechanism by which the brain dynamically allocates existing circuits to novel tasks, preserving latent computational mappings and enabling rapid restoration of original functions when the driving input subsides. Functional Precision, therefore, constitutes the dynamic basis by which neural networks sustain adaptability to shifting environments while safeguarding the coherence of their architectural design.

### 2.3 Evolutionary Precision: selection as the final editor of neural design

Across phylogenic timescales, Evolutionary Precision extends self-organizing principles, showing how natural selection sculpts not only survival-critical traits but also the internal efficiency of neural architectures. One possible compelling example of Evolutionary Precision under selective constraint is found in *Eigenmannia vicentespelea*, a blind, cave-dwelling electric fish. When its surface-dwelling ancestors became isolated in caves less than 20,000 years ago, the visual system quickly lost relevance (Fortune et al., 2020). Yet instead of fading spontaneously, the nervous system reorganized around a new sensory priority: electrosensation. Over generations, natural selection favored fish with stronger electric organ discharges (EODs), larger electric organs, and more flexible signal patterns, traits that enhanced electrolocation and electrocommunication in complete darkness. Selection acted on inherited neural traits that maintained coherence between sensory input and behavior under new environmental conditions. What began as functional re-weighting eventually stabilized as inherited neural architecture (Fortune et al., 2020).

Such a process reflects evolutionary self-organization, where local neural interactions converge on stable, adaptive configurations without central control. Underlying rules filter out noise, reinforce consistency, and refine structure. In *E. vicentespelea*, the electrosensory network did not emerge by chance; it arose via distributed adjustments that strengthened relevant pathways while de-emphasizing unused ones (Soares et al., 2023). Reiterated across generations, this consistent filtering produces a directional selection pressure that shapes circuitry. This aligns with multimodular plasticity: the nervous system reused and scaled pre-existing motor and sensory modules rather than building new circuits. Vision regressed not arbitrarily but because its computational role was no longer useful, a form of Precision through removal.

Put simply, the cavefish’s brain did not become more complex; it became more coherent. Precision acted as an organizing force, guiding evolution toward efficient, modular reuse attuned to persistent darkness. Crucially, this is not Lamarckian: use does not strengthen traits across generations. Random mutation supplies variation, and non-random selection in an aphotic environment favors variants that improve nonvisual sensing, giving a long-run appearance of reinforcement. Within-lifetime recalibration sets context, but only genetic changes that support it are retained and, across many generations, encoded. Though Evolutionary Precision unfolds on the longest timescales, it does not stand above the others; it ultimately crystallizes what Structural and Functional Precision continually generate. Processes like these underscores the need for a general formalism capable of capturing how coherence is preserved across structural, functional, and evolutionary scales. Together, the three are truly co-emergent expressions of a single logic, each shaping, constraining, and refining the others in a recursive dance of adaptation and coherence.

### 2.4 The Precision Coefficient: a quantitative outline of the *Precision Principle*

To make the *Precision Principle* more concrete without turning it into a full optimization routine, we introduce a quantitative expression that we call the *Precision Coefficient*, $P\left(z\right)$ . This coefficient balances network coherence against resource demands in a simple, heuristic way. We present the coefficient as anempirical balance that expresses the theory’s coherence. We present the coefficient as an empirical balance that expresses the theory’s coherence-versus-cost logic, not as an objective the brain is presumed to optimize. Formally, we define it as follows Equation 3:

$$P\left(z\right)=C\left(z\right)\;–\alpha \;R\left(z\right),\alpha \geq 0$$ (3)

This equation defines the *Precision Coefficient*, $P\left(z\right)$ , as a balance between coherence and cost. Here, z represents the set of long-term synaptic weights, and α is a trade-off parameter that determines how strongly resource conservation is weighted. The coherence term is written as, Equation 4:

$$C\left(z\right)=w_{Q}Q\left(z\right)+w_{E}E\left(z\right)+w_{\text{Cl}}\text{Cl}\left(z\right)+w_{\text{HC}}\text{HC}\left(z\right)$$ (4)

where $Q\left(z\right)$ is modularity, $E\left(z\right)$ is global efficiency, $Cl\left(z\right)$ is clustering, and $HC\left(z\right)$ is hub centrality. Each factor is scaled by non-negative weights, *w,* with all weights summing to one, reflecting their relative contributions to overall network coherence. The resource term is written as, Equation 5:

$$R\left(z\right)=v_{\text{WC}}\;\text{WC}\left(z\right)+v_{\text{Red}}\;\text{Red}\left(z\right)+v_{\text{Met}}\;\text{Met}\left(z\right)$$ (5)

where $WC\left(z\right)$ captures wiring cost, $Red\left(z\right)$ captures structural redundancy, and $Met\left(z\right)$ reflects metabolic load. Each term is scaled by a non-negative weight v , with all weights summing to one. Taken together, the *Precision Coefficient* increases when the network raises modularity, efficiency, clustering, and hub centrality without incurring excess wiring, redundancy, or metabolic load. Smaller values of α favor integration and complexity, whereas larger values emphasize economy and conservation.

In practice, the coefficient can be read operationally as a driver of learning. When activity updates tend to increase $P\left(z\right)$ , Hebbian reinforcement expands informative pathways, competitive dynamics suppress weaker ones, reinforcement consolidates high-value structure, and top-down matching gates when updates occur (Dresp-Langley, 2024). In this sense, $P\left(z\right)$ serves as a compact ledger of synaptic selection under constraint.

This interpretation is consistent with established neural learning equations. Grossberg’s long-term memory rule (Equation 1) describes how persistent synaptic weights $z_{ji}$ evolve through selective reinforcement and decay, providing a substrate for Structural Precision, while short-term memory dynamics (Equation 2) capture transient reconfiguration that supports Functional Precision (Grossberg, 1993). The coefficient summarizes these mechanisms within a single coherence-cost balance. At the network level, circuits conserve internal coherence by pruning weaker links and strengthening effective ones while exporting entropy to the environment. Together, these dynamics exemplify what we call precision coding: the principle that neural systems optimize representational efficiency by balancing coherence with flexibility.

Conceptually, precision coding is an allocation rule on limited representational capacity. Hebbian correlation makes candidate features locally available; competition (winner-take-all or k-winners) selects the active subset; and resonance or template matching stabilizes categories when top-down and bottom-up signals agree (Dresp-Langley, 2024). Increases in $C\left(z\right)$ correspond to sparser and more informative assemblies; decreases in $R\left(z\right)$ correspond to leaner codebooks and shorter, less redundant routes. For a fixed trade-off parameter α , improvements in $P\left(z\right)$ should coincide with higher activity sparsity, sharper community boundaries in connectivity, and a contraction of the effective dimensionality of population responses (Dresp-Langley, 2024).

To make the role of the Precision Coefficient concrete, we outline representative learning mechanisms that map changes in synaptic strength, competition, and connectivity to $C\left(z\right)$ , $R\left(z\right)$ , and their balance in $P\left(z\right)$ , as summarized in Table 2:

**TABLE 2:** Summary of representative neural learning mechanisms and their effects on the Precision Coefficient (Dresp-Langley, 2024).

Mechanism	Network effect	Effect on (P(z))
Hebbian LTP/LTD	Strengthens consistently co-active synapses and weakens inconsistent ones, improving the signal-to-noise ratio of pathways	Stabilization of reliable links increases C(z), pruning reduces R(z), and together P(z) rises
k-winners/WTA competition	Enforces sparsity by allowing only the strongest responses to persist, forming distinct codes and sharper module boundaries	More selective structure increases C(z)while redundant links are cut from R(z), resulting in a higher P(z)
Reinforcement learning	Consolidates pathways associated with predictive or rewarding outcomes while suppressing low-value alternatives	Selective reinforcement increases C(z), and with competition reduces R(z), driving P(z) upward
Modular connectivity	Builds small-world architecture with cohesive modules and targeted long-range bridges, supported by hub formation	Efficiency and integration increase C(z) without proportional growth in wiring cost, elevating P(z)
Plasticity and reorganization	Remaps circuits under new inputs or tasks, trimming unused synapses and scaling connections to maintain balance	Prevents R(z) from rising while preserving or restoring C(z), which maintains or improves P(z)
Receptive-field hierarchy	Combines local features into progressively more integrated and invariant representations across layers	Integration and clustering expand C(z) with modest increases in R(z), producing gradual growth in P(z)
ART resonance and matching	Allows updates only when top-down templates match bottom-up input, preventing drift and stabilizing learned categories	Learning is gated to conditions of high C(z) while limiting unnecessary growth in R(z), which increases P(z)

### 2.5 The core operations: a mechanistic basis of precision

**FIGURE 2:**
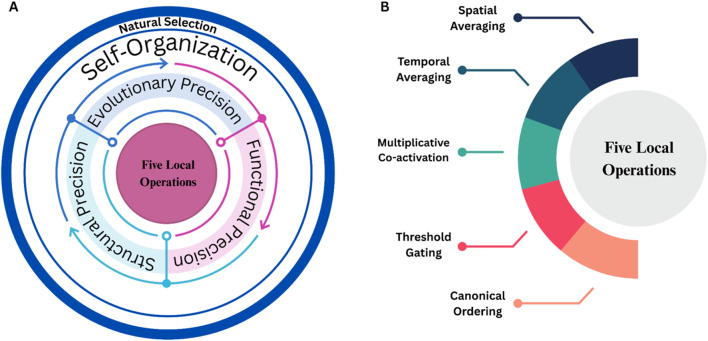
Panel **(A)** illustrates the overall recursive framework. At its center are five local operations, which both give rise to and are continuously shaped by three parallel “precision” arenas (middle annuli): Structural Precision, Functional Precision, Evolutionary Precision. Together, these form the *Precision Principle*. Panel **(B)** highlights these five core operations, spatial averaging, temporal averaging, multiplicative co-activation, threshold gating, and canonical ordering. This depiction avoids hierarchy, as no single form of Precision contains the others, and it preserves recursion: local processes generate Precision, precision generates self-organization, self-organization yields variation, and natural selection acts upon that variation which in turn influences the local rules over generations.

To illustrate the mechanistic basis of the *Precision Principle*, we propose Figure 2. Therein, five core local operations are identified: Spatial Averaging, Temporal Averaging, Multiplicative Co-activation, Threshold Gating, and Canonical Ordering. These enable the emergence of adaptive, cluster-rich network architectures. Together, these five operations form the universal substrate across *Structural, Functional*, and Evolutionary Precision.

According to the *Free Energy Principle* (*FEP*), the brain functions as a surprise-minimizing engine: perception, action, and learning are all directed toward reducing prediction error, and any neural reconfiguration follows wherever free energy can be lowered (Friston, 2010). Presenting a contrasting logic for brain self-organization, the *Precision Principle* suggests that the brain is intrinsically compelled to preserve and refine its own circuitry. Prediction errors serve as targeted prompts within this broader structural refinement process rather than the sole guiding force. At its core, the deeper imperative of the *Precision Principle* is to maintain circuit coherence, an ongoing process that ensures neural networks remain economical, modular, and easily reusable.

Underlying mechanisms of this intrinsic self-organization unfold through molecular and developmental programs. Neuropeptide-gated pruning during critical periods, time-locked growth and stabilization phases, and long-term consolidation dynamics all contribute to actively streamlining connections and aligning network hubs to patterns of input. Refinement driven by coherence proceeds even when salient prediction errors are absent, allowing for elegant microcircuits and hierarchical modules to emerge reliably across individuals and species (Chatterjee and Zielinski, 2020).

Viewed from this perspective, behavior extends beyond mere mismatch avoidance with the external world. It also reinforces patterns that safeguard internal network integrity (D’Angiulli and Roy, 2024). Actions that stabilize coherent mappings grow increasingly valuable, further entrenching efficient architectures (D’Angiulli and Roy, 2024). Centering intrinsic circuit coherence as the key engine of self-organization, not just surprise minimization, the *Precision Principle* clarifies how rapid adaptation, developmental sensitivity, and evolutionary stability all arise from the brain’s internal structural logic.

## 3 Discussions and limitations

While the *Precision Principle *deliberately avoids conflating biological self-organization with conscious intention, more work is needed to define how it interacts with higher-order phenomena such as imagination, reflection, and volitional planning. The proposed mechanism as discussed in this paper, likely operates at multiple nested scales, from local microcircuits up to large-scale brain networks, with each of the five local operations tuned differently depending on the level of processing. In the case of primary sensory areas, spatial and temporal averaging windows may be narrow, sharpening feature detection, whereas in associative and prefrontal regions, broader windows could support the integration of diverse inputs over longer time spans, enabling sustained deliberation and planning (Chaudhuri et al., 2015). Exploring how these multi-scale precision parameters develop over childhood or become altered in conditions such as autism or schizophrenia could yield new biomarkers for cognitive flexibility and rigidity.

While the *Precision Coefficient* provides a compact expression of the balance between coherence and cost, it should be regarded as a generative lens rather than a closed optimization program. Its strength lies in clarifying how different pressures interact, but its limitations must also be explicit. The precise value of $P\left(z\right)$ depends on how coherence and cost are measured, and on the context-specific choice of the trade-off parameter α . Different yet equally valid metrics for modularity, efficiency, clustering, hub centrality, wiring cost, redundancy, or metabolic load will shift numerical outcomes without altering the principle itself. Moreover, any attempt to estimate $P\left(z\right)$ empirically will face issues of noise, non-identifiability, and sensitivity to parameterization. These caveats do not weaken the framework; rather, they emphasize that the coefficient is best understood as a unifying measure of constraint-driven coherence across scales, not as evidence that the brain is solving a single fixed optimization problem. Accordingly, we call for a reproducible evaluation pipeline that varies α and metric definitions, tests inference stability under realistic noise and sampling regimes, and reports predictive accuracy against withheld data so that acceptance or revision of the model is driven by prospective performance rather than *post hoc* fit.

Another important condition concerns dissipation, since neural circuits cannot sustain coherence without exporting entropy and consuming energy. The resource term must therefore reflect not only structural wiring costs but also the metabolic expenditure needed to maintain activity. In our formulation, dissipation falls under the metabolic component of *R (z)*, yet in practice it should be tracked explicitly through physiological measures such as glucose uptake, oxygen use, or hemodynamic response. This ensures that increases in coherence that require disproportionate energy will reduce *P (z)*, keeping the coefficient grounded in biological feasibility rather than abstract topology.

Building on this, models of perception should capitalize on biologically grounded self-organization to reduce explicit structural complexity while improving both Structural and Functional Precision, letting robustness arise from the interaction of simple local rules rather than from ever-deeper engineered architectures. For example, the Digital Hormone Model (DHM) simulates how uniform skin cells can self-organize into complex, biologically accurate patterns such as feather buds, which are the early developmental structures from which feathers grow in birds. These buds emerge in precise spatial arrangements not through centralized control, but via local hormone-like signaling between neighboring cells. The model demonstrates how simple, biologically inspired rules can generate functionally robust and structurally organized outcomes, illustrating the power of self-organization over engineered complexity (Shen and Chuong, 2002).

Additionally, while the five core local operations offer a useful minimal scaffold, several cautions must be considered. Much of the supporting evidence comes from *in vitro* preparations with limited neuromodulation and constrained inputs, which can artificially inflate apparent stability due to spatial and temporal averaging (Orlandi et al., 2014; Shew et al., 2009). Reports of modular, small-world, and near-critical organization are sensitive to thresholding, temporal binning, and finite-size effects, so parametric sweeps and surrogate-data tests are needed to separate biology from analysis artefact (Gireesh and Plenz, 2008; Klaus et al., 2011). Coincidence-based potentiation and gating can discard weak-but-predictive signals or sequence codes, and *in vivo* they are likely tempered by state-dependent control and homeostatic regulation (Mizraji and Lin, 2015; Shen and Loew, 2016; Elliott, 2024). Finally, a single canonical ordering may not capture laminar, cell-type, or behavioural-state differences in decay/gating/potentiation kinetics (Elliott, 2024). Overall, the operations remain a valuable constraint set, but their boundary conditions should be explicit and tested across states, timescales, and analysis choices.

As self-organization illustrates across disciplines, solutions discovered in one context can inform another. This perspective therefore aims to guide future work in perceptual research and artificial intelligence: favor parsimonious, self-organizing mechanisms with well-specified boundary conditions over highly elaborate constructions whose functional resilience is uncertain, so that complexity emerges from dynamics rather than design. Within neural systems, this same logic is implemented by neuromodulators that regulate when and how plasticity is expressed. For example, transient bursts of acetylcholine or dopamine can lower the threshold for potentiation and widen temporal averaging during learning episodes, admitting novel patterns into the network, and then restore stricter gating and faster decay during consolidation to protect established assemblies (Zannone et al., 2018). Dynamic modulation in this sense links precision to arousal, attention, and motivation, and it predicts that pharmacological or optogenetic manipulation of neuromodulators will shift the balance between plasticity and stability in systematic ways (Muir et al., 2024). Viewed through the lens of the coefficient, such surges can be read as temporary adjustments of the effective α, tilting the system between integration and economy across learning, consolidation, and arousal, thereby anchoring biochemical control to the coherence–cost equilibrium formalized in this paper.

At a conceptual level, the mechanistic foundations of the *Precision Principle* intersect with broader debates on the role of information not only in neural systems but as the building blocks of reality, and this framing sets the stage for how precision governs what gets connected and kept. The “information-as-power” model is a naïve view of information as a linear route to truth and control, an objective and representative of reality. In contrast, an enlightened perspective emphasizes information as a connector valued for sustaining meaningful relationships within a system (Harari, 2024), preparing the ground for a formal account of how such relationships are weighted and updated.

Building on this relational view, embedding the *Precision Principle* within active-inference frameworks recasts those five local operations as implementations of confidence weighting in predictive coding. Spatial averaging aggregates prediction errors across feature channels (Sweeny et al., 2009), while temporal averaging corresponds to the autocorrelation of error signals over time (Stier et al., 2025). Volitional planning and reflective thought can then be seen as top-down adjustments of these confidence weights, selectively amplifying or dampening error signals to guide belief updating toward desired goals. Empirical tests might therefore pair electrophysiological markers of neural precision with behavioral measures of decision confidence, leading directly to the biological operations that realize these weighting processes.

At the biological level, reinforcement and pruning become the concrete operations that determine when a synapse earns persistence. Correlated spiking under permissive modulatory state consolidates a subset of weights, while local inhibitory circuits and synaptic scaling impose competition by normalizing total excitatory drive so that only strongly supported pathways remain. Glial mechanisms contribute further by removing low-utility spines when activity chronically falls below threshold (Schafer et al., 2012). In the coefficient formalism, these operations add positive change to coherence when they stabilize paths that shorten communicability and strengthen integrative hubs, and they subtract from resource cost when they reduce wiring length and duplicate routes. Over-elimination can depress coherence even as cost falls, so homeostatic rules restore the balance that keeps net change in the coefficient non-negative under stable demands. The outcome is a family of metastable, high-coherence configurations that can be re-entered after perturbation, which provides the foundation for a systems-level reinterpretation of brain function.

Viewed through this lens, the brain is better described as a connection-maximizer than as a truth-maximizer. From this perspective, learning is not merely the correction of prediction errors but a process of connective rebalancing. Information is therefore not retained for external accuracy alone but because its incorporation actively reinforces and reshapes the functional architecture of our brains. Extending beyond neurobiology, one can imagine computational models that evaluate incoming data not by reward signals alone but by an “integration gain” metric, measuring how much each update improves network topology (modularity, hub centrality, small-worldness), thereby linking biological coherence to formal design principles for artificial systems.

Translating the *Precision Principle* into neuromorphic hardware then becomes a natural step, using leaky integrator circuits for temporal averaging, programmable comparators for threshold gating, and coincidence-detecting synapses for multiplicative co-activation, all cycling through ordered decay and potentiation phases (Indiveri et al., 2011; Davies et al., 2018; Prezioso et al., 2018). Embedded in silicon, these processes could yield processors that self-organize into efficient, robust architectures, capable of adapting under noisy conditions while consuming minimal power. In this way, the hardware instantiates the full pipeline from relational information to confidence weighting to coherence-preserving plasticity, realizing the *Precision Principle* as a principle of efficient and resilient self-organization.

Taken together, these considerations suggest that the *Precision Coefficient* should be regarded not as a narrow formula but as an expression of the *Precision Principle’s* role as a driver of neural learning. It foregrounds the interplay of coherence, dissipation, and selective elimination, showing how local mechanisms such as Hebbian reinforcement, k-winners competitive selection, and the elimination of weaker synaptic connections scale upward to organize networks and, in due course, entire brains.

Ultimately, the *Precision Principle* as proposed in this paper transcends mere wiring rules, emerging as a scale-invariant universal force that sculpts coherence at all dimensions. By filtering, gating, and temporally sculpting neural activity, the brain does not just record reality; it architects its own structure, demanding that every spark of imagination, every strategic plan, every reflective thought earn its place by reinforcing the network’s integrity. In this light, precision is not passive order-taking but the active crucible in which noise is forged into narratives, randomness into reason, and fleeting sparks into enduring circuitry.

## Data Availability

The original contributions presented in the study are included in the article/supplementary material, further inquiries can be directed to the corresponding authors.
